# Divergent evolution in the cytoplasmic domains of PRLR and GHR genes in Artiodactyla

**DOI:** 10.1186/1471-2148-9-172

**Published:** 2009-07-22

**Authors:** Terhi Iso-Touru, Juha Kantanen, Meng-Hua Li, Zygmunt Gizejewski, Johanna Vilkki

**Affiliations:** 1 Biotechnology and Food Research, MTT Agrifood Research Finland, 31600 Jokioinen, Finland; 2 Institute of Animal Reproduction and Food Research, Polish Academy of Sciences, Pl-10-747 Olsztyn, Tuwima 10, Poland; 3 Ecological Genetics Research Unit, Department of Biological and Environmental Sciences, PO Box 65, FI-00014 University of Helsinki, Finland

## Abstract

**Background:**

Prolactin receptor (PRLR) and growth hormone receptor (GHR) belong to the large superfamily of class 1 cytokine receptors. Both of them have been identified as candidate genes affecting key quantitative traits, like growth and reproduction in livestock. We have previously studied the molecular anatomy of the cytoplasmic domain of GHR in different cattle breeds and artiodactyl species. In this study we have analysed the corresponding cytoplasmic signalling region of PRLR.

**Results:**

We sequenced PRLR gene exon 10, coding for the major part of the cytoplasmic domain, from cattle, American bison, European bison, yak, sheep, pig and wild boar individuals. We found different patterns of variation in the two receptors within and between ruminants and pigs. Pigs and bison species have no variation within GHR exon 10, but show high haplotype diversity for the PRLR exon 10. In cattle, PRLR shows lower diversity than GHR. The Bovinae PRLR haplotype network fits better the known phylogenetic relationships between the species than that of the GHR, where differences within cattle breeds are larger than between the different species in the subfamily. By comparison with the wild boar haplotypes, a high number of subsequent nonsynonymous substitutions seem to have accumulated in the pig PRLR exon 10 after domestication.

**Conclusion:**

Both genes affect a multitude of traits that have been targets of selection after domestication. The genes seem to have responded differently to different selection pressures imposed by human artificial selection. The results suggest possible effects of selective sweeps in GHR before domestication in the pig lineage or species divergence in the Bison lineage. The PRLR results may be explained by strong directional selection in pigs or functional switching.

## Background

Domestication or the unique form of mutualism that develops between a human population and a target animal population has strong selective advantages for both partners [1]. Livestock domestication started around 10000 years ago in the Fertile Crescent, beginning with goats and sheep [2]. Important traits regarding domestication include behaviour and reproduction as well as dairying [3]. Milking of the ruminant animals was practiced intensively already over 8000 years ago (sixth and seventh millennia BC) in northwest Anatolia. Domesticated livestock species have undergone and are constantly under artificial selection. Strong directional selection in domestic animals is postulated to have led to selective sweeps in which alleles at loci that underlie selected traits (growth, fertility, milk production, coat colour) have decreased or increased markedly in their frequency [4]. A recent genome wide analysis of cattle identified detectable signatures of domestication and artificial selection in the cattle genome, but also that the current levels of diversity within breeds are at least as great as within humans [5]. However, the effects of domestication and artificial selection are still mostly unknown at the level of nucleotide sequence variation.

Accumulating knowledge of the domestic species genomes has enabled mapping of loci affecting key traits under artificial selection in livestock. In cattle, numerous such quantitative trait loci (QTL) have been identified. Over fifty QTLs have been localized to cattle chromosome 20 . Two interesting candidate genes with potential effects on various agronomically important traits, prolactin receptor (PRLR) and growth hormone receptor (GHR) locate in this chromosome at a distance of 7.5 Mb (Btau4.0, Oct.2007, ). An S to N substitution (S18N) in the signal peptide of the PRLR is linked with protein and fat yield [6] and the substitution F279N in the transmembrane part of the GHR has been suggested to be a quantitative trait nucleotide/causative mutation affecting milk fat and protein percentage [7].

Both GHR and PRLR belong to the superfamily of class I cytokine receptors, which presumably arose as the result of multiple gene duplications and subsequent divergent evolution. GHR and PRLR share a common tertiary structure (an extracellular domain, a single membrane spanning transmembrane domain, and a cytoplasmic domain). They mediate the signals of their ligands, the growth hormone (GH), prolactin (PRL) and placental lactogen (PL). The binding of the ligand to the extracellular part induces homo- or heterodimerization of the receptors, followed by intracellular signal transmission by the JAK-Stat signalling pathway [8,9]. Both GHR and PRLR are involved in mammary growth and function. The main biological role of the GH is the control of postnatal growth, whereas the additional reported effects of PRL include involvement in seasonality, reproduction, behaviour and immunoregulation. Amino acid sequence identity between GHR and PRLR varies between 30% and 70% depending on the part of the receptor. The greatest similarity of the aa sequences between GHR and PRLR is in the extracellular domains. The cytoplasmic domain and especially the well conserved BOX1 is essential for signal transduction.

We have previously examined the GHR cytoplasmic domain sequence in different cattle breeds and Artiodactyla species [10], where we observed interesting polymorphism. The aim of this study was to characterize patterns of polymorphism in the corresponding intracellular region of PRLR in different Artiodactyla species and breeds to enable the comparison of sequence evolution in response to domestication and artificial selection in two evolutionary related and closely located candidate genes.

## Results

**Figure 1:**
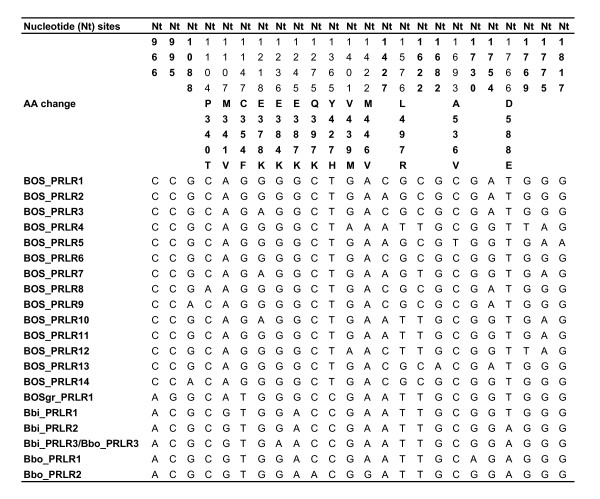
**Statistically inferred cattle, yak, American bison and European bison haplotypes from the PRLR exon 10**. Only variable sites are shown. SNP numbering is based on the sequence NM_001039726.1. Amino acid numbering starts from the first methionine in the protein sequence NP_001034815.1.

The sequence analysis of the cytoplasmic domain of the PRLR gene revealed interesting variation in wild and domesticated Artiodactyla species and subspecies (Table 1). Altogether 13 SNPs were found from the cattle samples. Of these six were present in both European and African cattle: one nsSNP (E378K) and five sSNPs (Nt1088, Nt1427, Nt1622, Nt1754, and Nt1775). European cattle had two private nsSNPs (P340T, A536V) and two private sSNPs (Nt1682, Nt1817) while African cattle had two private nsSNPs (V439M and L497R) and one private sSNP (Nt1769). The cattle SNPs were inferred to segregate as 14 haplotypes (Figure 1) two of which were shared by European and African cattle (haplotypes BOS_PRLR1 and BOS_PRLR2). American bison and European bison shared one nsSNP (E384K). Besides that, American bison had one private nsSNP (D588E). European bison samples were more divergent; they had two private nsSNPs (Q397K and M446V) and one sSNP (Nt1730). Statistically inferred haplotypes for the American bison and European bison are given in Figure 1. American bison and European bison share one haplotype, BBI_PRLR3/BBO_PRLR3. The studied yak samples were monomorphic (Figure 1). Sheep samples carried four sSNP (Nt1007, Nt1160, Nt1217, and Nt1400) and three nsSNP (E387K, A476T, and S480R) in exon 10. The E387K position has been fixed to E in the Bos lineage and K in the Bison lineage.

**Table 1:** Diversity and neutrality indices in exon 10 of PRLR and GHR genes in the studied species.

Gene	Species			No. of		No. of				Tajima's	Fu and Li's	
		n	lenght (bp)	ns	s	haplotypes	H_d_	π^±^	θ^±^	D	D*	F*
PRLR	European Cattle (*Bos taurus*)	216	891	3	7	9	0.44	0.84	1.69	-1.08	-0.41	-0.8
	African Cattle (*Bos indicus)*	22	891	3	6	7	0.76	3.98	2.32	2.05*	1.36	1.86*
	Yak *(Bos grunniens)*	4	891			1						
	American bison *(Bison bison)*	3	891	2	0	3	0.73	1.05	0.98	0.31	0.06	0.12
	European bison *(Bison bonasus)*	5	891	3	1	3	0.73	2.17	1.59	1.41	1.24	1.43
	Sheep *(Ovis aries)*	14	891	3	4	6	0.61	1.18	1.4	-0.47	-0.78	-0.8
	Domestic pig (*Sus scrofa*)	18	750	8	1	5	0.63	3.27	2.89	0.39	1.36	1.24
	Wild boar (*Sus Scrofa*)	9	750	1	1	2^∞^	0.21	0.56	0.78	-0.68	0.88	0.53

GHR	European Cattle (*Bos taurus*)	202	900	4	3	18	0.69	2.07	1.18	1.47	1.09	1.47
	African Cattle (*Bos indicus)*	47	900	4	6	7	0.72	3.97	2.17	2.13*	1.38	1.94*
	Yak (Bos grunniens)	4	900			1						
	American bison (*Bison bison*)	3	900			1						
	European bison *(Bison bonasus)*	6	900			1						
	Sheep *(Ovis aries)*	14	900	3		4	0.66	1.18	0.86	0.92	0.96	1.1
	Domestic pig (*Sus scrofa*)	18	690			1						
	Wild boar (*Sus Scrofa*)	9	690			1						

European cattle breeds are grouped and African cattle breeds are grouped. ns = nsSNP, s = sSNP, H_d _= haplotype diversity, π = nucleotide diversity, θ_W _= Watterson's theta estimator, * P < 0.05, ^± ^per kilobase between sequences, ^∞^does not include SUS_PRLRL4_del haplotype

**Figure 2:**
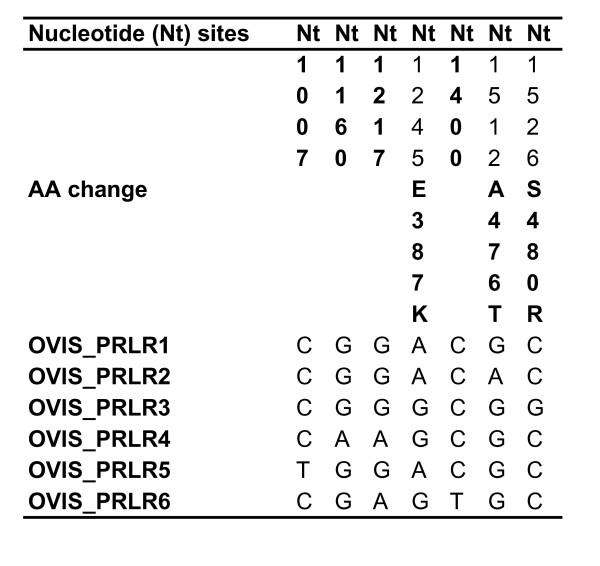
**Statistically inferred sheep haplotypes from the PRLR exon 10**.

Statistical haplotype reconstruction uncovered 4 different sheep haplotypes. Cloning of one ambiguous sheep individual revealed two unique haplotypes (OVIS_PRLR5 and OVIS_PRLR6) and these haplotypes were included in further analysis (Figure 2). Numbering of the *Bos *and *Bison*, and *Ovis *SNPs is based on the reference sequence NM_001039726. All *Bos*, *Bison *and *Ovis *SNPs were in Hardy-Weinberg equilibrium.

**Figure 3:**
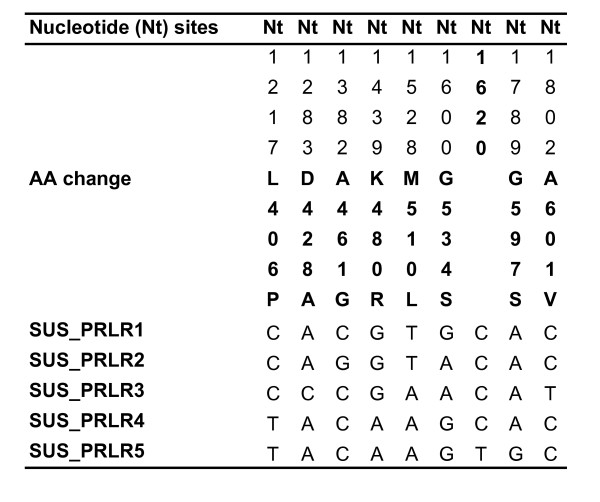
**Statistically inferred pig haplotypes from the PRLR exon 10**.

From the domestic pig samples we found one sSNP (Nt1620) and eight nsSNPs (L406P, D428A, A461G, K480R, M510L, G534S, G597S, and A601V). Numbering of the pig SNPs is based on the pig cDNA sequence DQ157757, according to the numbering of previously identified SNPs [11]. All SNPs were in Hardy-Weinberg equilibrium. The SNPs in pig exon 10 constitute 5 different haplotypes (Figure 3). Seven out of nine wild boar individuals were SUS_PRLR4 homozygotes, one individual was SUS_PRLR5 homozygote and one individual had haplotype SUS_PRLR4 and haplotype SUS_PRLR4-del. SUS_PRLR4-del haplotype has a three-nucleotide deletion (Nt1439 – Nt1441) inducing lack of aa 480. PRLR haplotype sequences from the different species have been deposited in GenBank  with the accession numbers FJ901275 – FJ901301 and FJ901303 – FJ901307.

Comparison of variation in the GHR and PRLR cytoplasmic domains in the species analysed in both studies is presented in Table 1. New results from the wild boar and European bison GHR sequencing have been added. All wild boars were monomorphic having the same haplotype in GHR exon 10 as domestic pigs (FJ901302) and European bison was shown to have the same GHR exon 10 haplotype [GenBank:DQ062723] as American bison. The most dramatic difference in the variation between GHR and PRLR within species is seen in the domestic pig. The complete lack of variation in GHR is contrasted with the high haplotype and nucleotide diversity in the PRLR, consisting of mainly nonsynonymous variation.

In the Bovinae subfamily PRLR and GHR show different features in the two *Bison *species from cattle. The *Bison *species, like pigs, are monomorphic for GHR; but show high level of haplotype diversity in PRLR. The African cattle shows equally high levels of diversity at both genes, whereas in European cattle the haplotype and nucleotide diversity is lower at PRLR. The yak is monomorphic for both genes. Sheep belongs to the same family, Bovidae, as Bovinae species. Sheep has similar number of nsSNPs in GHR as in PRLR; however PRLR in all is more polymorphic.

The different indices of neutrality at PRLR, Tajima's D value, Fu and Li's D* and F* values are mainly reflecting the same pattern with each other. The D values for African cattle, European bison and domestic pig are positive and for European cattle and sheep negative (Table 1). Only the values for African cattle (both at PRLR and GHR) deviate statistically significantly from zero. Artificially selected populations, like livestock species, do not fulfil the assumptions of random mating and constant population size for the neutrality test, hence positive Tajima's D values are likely due to the demographic histories of these species or breeds rather than true balancing selection. Considering the known history of zebu-taurine crossbreeding in Africa [12], the observed allelic diversity is most likely caused by admixture

**Figure 4:**
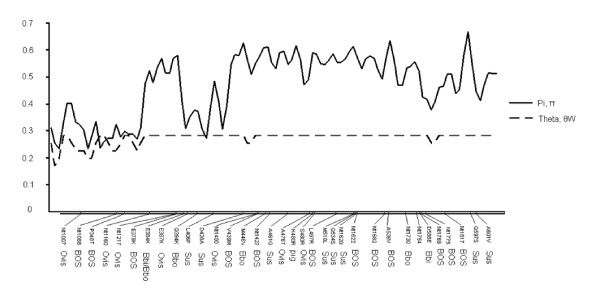
**Sliding window presentation of the nucleotide divergence along PRLR exon 10 among 18 different species**. Bta = cattle, Bbi = American bison, Bbo = European bison, Ovis = sheep, Sus = pig. The positions of the detected SNPs are shown on the X-axis.

A sliding window plot of the nucleotide divergence along PRLR exon 10 in 18 vertebrate species is presented in Figure 4. Two regions of low nucleotide diversity are evident, one at the beginning and one in the region between 270 bp and 400 bp from the beginning of the exon 10. All E to K amino acid changes in Bovidae (cattle E378K, American/European bison E384K, and sheep E387K) are within 27 bp in the second low diversity module and additionally one European bison K to Q substitution and two pig substitutions (L406P and D428A) fall inside this region. We studied the possible impacts of the aa changes for the protein structure with the methods implemented in SIFT and PolyPhen programs [13,14]. The substitution E378K found in cattle was predicted to affect protein function by SIFT analysis (score 0.02). Two other SNPs, E384K in both bison species and V439M in cattle, got SIFT scores below 0.1 (Table 2). This cut-off value has been suggested to provide better sensitivity for detecting deleterious SNPs [13]. E (glutamic acid) is a polar, acidic amino acid; K (lysine) is a polar and basic amino acid; V (valine) and M (methionine) are nonpolar and neutral amino acids.

**Table 2:** Predicted affection status for the amino acid substitutions from PRLR gene exon 10

	SIFT				PolyPhen		
Species	prediction	score	MSC	n	prediction	PSIC^±^	n
European Cattle (*Bos taurus*)							
P340T	tolerated	0.35	3.1	30	benign	0.78	33
E378K	affect protein function	0.02	3.12	28	benign	0.88	27
A536V	tolerated	0.34	3.1	29	benign	0.41	31
African Cattle (*Bos indicus)*							
V439M	tolerated	0.07	3.1	29	benign	0.39	31
L497R	tolerated	0.36	3.1	29	benign	0.13	30
American bison (*Bison bison*)							
E384K*	tolerated	0.06	3.08	27	benign	1.48	23
European bison *(Bison bonasus)*							
E384K*	tolerated	0.06	3.05	29	benign	1.50	22
Q394K*	tolerated	0.16	3.05	29	benign	1.21	24
M445V*	tolerated	0.42	3.05	28	benign	1.48	22
Sheep *(Ovis aries)*							
E387K	tolerated	0.47	3.05	29	benign	0.57	31
A476T	tolerated	0.64	3.05	29	benign	0.10	30
S480R	tolerated	0.62	3.05	29	benign	0.24	31
Domestic pig (*Sus scrofa*)							
L406P	tolerated	0.25	3.16	27	benign	0.65	23
D428A	tolerated	0.54	3.15	29	benign	0.04	28
A461G	tolerated	0.36	3.15	29	benign	1.34	28
K480R	tolerated	0.61	3.15	29	benign	0.29	28
M510L	tolerated	0.64	3.15	29	benign	1.31	28
G534S	tolerated	0.79	3.17	25	benign	0.23	23
G597S	tolerated	0.65	3.15	25	benign	0.35	18
A601V	tolerated	0.23	3.15	25	benign	0.51	19

* analyses were done using the cytoplasmic part alone, MSC = median sequence conservation, n = number of sequences represent at this position in the protein alignment, ^± ^PSIC score difference

Linkage disequilibrium (LD) analysis revealed that in European cattle only one pairwise SNP comparison showed LD (r^2 ^= 1). On the other hand, in African cattle seven pairwise SNP comparisons were in LD (r^2 ^= 1). The same LD pattern was visible in GHR exon 10 [10]. In sheep one pairwise SNP comparison was in LD (r^2 ^= 1), in pigs two pairwise SNP comparisons showed LD (r^2 ^= 1).

**Figure 5:**
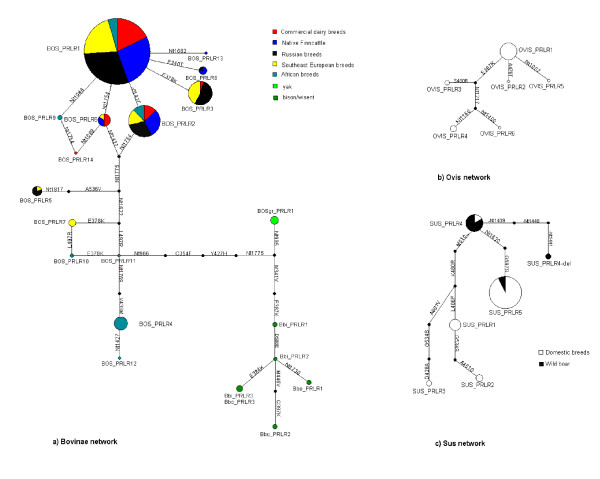
**Parsimony network reconstructions**. a) Bovinae haplotype network. Different colours represent different cattle breed groups and/or species. b) Ovis haplotype network c) Sus haplotype network. The size of the pie is proportional to the frequency of the haplotype for the breed group in question and the size of the node area is proportional to the total frequency of the haplotype in the whole population. Small black circles represent the hypothetical haplotypes not present in this study. BOS = cattle, Bbi = American bison, Bbo = European bison, BOSgr = yak, SUS = pig, OVIS = sheep.

A parsimony haplotype network for the subfamily Bovinae is presented in Figure 5a. Most of the common cattle haplotypes differ from the major haplotype BOS_PRLR1 only by one nucleotide. However, there are several deduced intermediate haplotypes that were not seen in this study (and some of the substitutions are present in two different locations on the network). The most common haplotype for the African cattle is BOS_PRLR4. It is absent in European cattle and differs by two nonsynonymous and five synonymous substitutions from the BOS_PRLR1 (the most common haplotype for the European cattle). Thus the BOS_PRLR4 as well as BOS_PRLR9, BOS_PRLR10, BOS_PRLR11 and BOS_PRLR12 probably represent zebu haplotypes in our data, reflecting the deep divergence between the taurine and zebu genomes [12]. According to the PRLR network, the yaks are more closely related to the genus Bison than to the genus Bos. The most frequent cattle haplotype BOS_PRLR1 and the yak haplotype BOSgr_PRLR1 differ by three nonsynonymous and by seven synonymous substitutions, whereas the yak haplotype differs from the American bison haplotype BBI_PRLR1 by three nonsynonymous substitutions. The haplotype BBI_PRLR1 differs by five nonsynonymous and by six synonymous substitutions from the BOS_PRLR1. Sheep haplotypes differ from each other with one or two substitutions, OVIS_PRLR1 being the major sheep haplotype (Figure 5b). Figure 5c presents the parsimony network from the Sus data. Substitution G534S is the only polymorphism that occurs twice in the network. The major wild boar haplotype SUS_PRLR4 is separated from the two most diverged domestic pig haplotypes SUS_PRLR2 and SUS_PRLR3 by five nonsynonymous substitutions.

## Discussion

In this study we demonstrate different patterns of variation in PRLR and GHR in ruminants and pigs, suggesting divergent evolution and selection pressures before and after domestication.

The PRLR molecular anatomy shows interesting differences from the GHR. The Bovinae PRLR haplotype network is very different from that of the GHR [10], where differences within European breeds, within African breeds and between European/African breeds were larger than between different species (cattle, yak, American bison as well European bison (data from European bison obtained from this study)). The PRLR network fits better to the known phylogenetic relationships between species. Within cattle breeds, European cattle are lacking major private PRLR haplotypes in contrast to the previous GHR study. Instead the two major haplotypes, BOS_PRLR1 and BOS_PRLR2 are shared between European and African cattle.

During pig domestication in Europe (started about 6000 years ago), domesticated progeny of the local European wild boars replaced introduced near eastern domestic pigs [15]. According to the coalescent theory the most frequent haplotype in a population level study is the ancestral haplotype [16], on the other hand Watterson et al [17] argued that the higher the mutation rate, the less likely it is that the most frequent allele would be the oldest. Considering the population history of the pigs, we assume that the major wild boar haplotype SUS_PRLR4, even though not the most frequent haplotype is a good candidate for being the ancestral haplotype of the European domestic pig. If true, substitutions leading to rest of the domestic pig Sus_PRLR haplotypes have happened after the domestication, as the haplotypes are derived by a series of consecutive substitutions. The result is similar with the result obtained from the MC1R gene that has an effect on coat colour in pigs [18]. MC1R alleles/haplotypes differ by more than one nonsynonymous mutation from the wild-type, implying a long history of strong positive selection for coat colour variants. It is argued that coat colour phenotypes result from direct human selection [18]. Three domestic pig haplotypes differ from the SUS_PRLR4 by two or more nonsynonymous substitutions, the largest difference being five nonsynonymous substitutions. The rapid accumulation of mutations in the PRLR gene in the pig is best explained by human influence. Domestication and following selection for agricultural purposes have reduced breed effective population sizes to relatively small numbers. In a population with low N_e _a larger fraction of slightly deleterious mutations can reach fixation due to less efficient purifying selection [19]. This may provide one explanation for the observed polymorphism in PRLR. However, the GHR gene is monomorphic in the same pig sample, suggesting different selection pressures (selective sweep or strong purifying selection in GHR, strong positive selection for PRLR).

After domestication the reproduction performance of the pig has increased several folds due to an increase in litters per year and piglets per litter [20]. This has been made possible by selection and making the environment more favourable for the sow to allocate resources to more offspring. Many studies in pigs have revealed significant association of PRLR polymorphisms with different reproduction traits (e.g. [11,21,22]). Mouse knockout models of the PRLR have confirmed the importance of the PRL in reproduction (reviewed by [23]). In addition to reproductive performance, the list of modes of actions of PRLR is long [8] and shows considerable change through vertebrate evolution [9].

If a gene has undergone repeated alternating functional adaptation to fulfil one or two, even more, functions, it can lead to accumulation of many amino acid substitutions with slightly little overall change of function. This phenomenon has been called functional switching [24] and could thus explain PRLR polymorphism. Based on the studies done with the domestic animals (e.g. [6,7,11,21,22,25]), PRLR could fulfil the criterion of functional switching. However, the accumulation of mutations in the domestic pig PRLR seems too fast to be explained by functional switching alone.

The major role of GHR involves postnatal growth and mammary function. The lack of variation in the GHR signalling domain in pigs and undomesticated ruminants is intriguing. The lack of variation in pigs could be the result of a strong selective sweep that has happened before domestication and has been maintained in domestic pigs. On the other hand, the low diversity in the wild boar may reflect a narrow genetic background of the Finnish wild boar population. The possible target (causative mutation) of selection at or close to the GHR exon 10 is not known. In contrast, the GHR gene cytoplasmic domain in current cattle breeds harbours lots of interesting persistent polymorphism, indicating its possible importance for cattle being malleable for artificial selection for growth and lactation traits [10].

The PRLR gene on the other hand shows contrasting evolution in the studied species, having more substitution polymorphism in pig breeds and bison species than in cattle. Functional analysis indicated possible effects for nsSNPs found in cattle and bison, but didn't reveal any effect on the protein structure for pig nsSNPs. However, methods defining effects of SNPs are only predictions, and the combined effects of various nsSNPs can not be analysed by current statistical methods. No known 3D structure for PRLR is available, so more detailed analysis whether the substitution sites are in spatial contact with critical residues remains unknown. Functional analysis at the molecular level would be necessary to solve the relevance of the polymorphisms.

Domestication is a cumulative process marked by changes on both sides of the mutualistic relationship. Long-living animals like cattle respond slower to selection than for example annual crops [1]. Traits that have been the target of domestication and subsequent selection and the genes that affect them, i.e. so called domestication genes, are of special interest even though the success to identify these genes from livestock has been low [1,4]. Genes identified by QTL studies are good candidates for genes important for domestication success [1].

## Conclusion

QTL candidate genes GHR and PRLR have varying roles in growth, lactation and reproduction in domestic species and may have responded differently in different species and breeds within species to different selection pressures. We show here that these genes have different evolutionary history among artiodactyls. Most likely both genes have been heavily influenced by artificial selection during and after domestication. We found an unanticipated amount of nonsynonymous variation accumulated or persisted in these genes in artiodactyl species during the short period of domestication (8000 – 10 000 years) and subsequent artificial selection. The differences between species indicate possible effects of selective sweeps before domestication (GHR in pigs) or before species divergence (GHR in *Bison*), directional (artificial) selection (PRLR in pigs) or functional switching (GHR in cattle, PRLR).

## Methods

### Materials

We investigated 216 individuals from 12 different European *Bos taurus *breeds (commercial dairy breeds: Finnish Ayrshire, Finnish Holstein-Friesian; native Finnish dairy breeds: Western Finncattle, Northern Finncattle, Eastern Finncattle, Russian breeds: Kholmogor, Yakut Cattle, Bestuzhev, and Belorussian Red, and Southeast European breeds: Busa, Podolian, and Ukrainian Grey). In addition, we studied 22 samples from 3 different African breeds from Ethiopia with unknown *Bos indicus *background (Barka; morphological zebu-type, Raya; morphological sanga-type, and Fogera; zebu-sanga intermediate). Cattle samples originated from 6 different countries (Finland, Russia, Ukraine, Byelorussia, Serbia, and Ethiopia). As references we studied 3 American bison individuals (*Bison bison*) from one breeding stock, 6 European bison (*Bison bonasus*) samples from Poland, and 4 yak (*Bos grunniens*) samples from Russia. Other studied species included 14 sheep (*Ovis aries*) representing 6 different breeds from Russia, Poland and Finland (Romanov breed, Wrzosowka breed, Dagestan local, Andi, sheep from Komi village, Finnsheep, and Ålandsheep), 18 pigs (*Sus scrofa*) representing Large White, Landrace, Hampshire and Duroc breeds, and 9 wild boar samples (*Sus scrofa*) from three different Finnish wild boar farms. Individuals from the same breed/species were sampled to be as unrelated as possible. European bison is an endangered species and all current animals (approximately 3200 individuals) descend from 12 founder animals [26]. All the studied species belong to the same order, Artiodactyla. Pig belongs to the family Suidae, while all other species belong to the family Bovidae. Cattle and yak are from the genus Bos, while American and European bison belong to genus Bison.

### Genetic analysis

Genomic DNA was extracted from blood or semen samples using salting out procedure [27]. The prolactin receptor gene exon 10 was amplified in two fragments with the same primer sets from cattle, yak, and bison samples. Exon 10 from the pig and sheep samples was amplified in one fragment. Primers were designed with the Primer3  using reference sequences [GenBank: NM_001039726 and DQ458765.1]. The PRLR exon 10 sequence of the ruminant comprises of 891 bp (297 aa), which was sequenced entirely, and that of the pig is 1023 bp (341 aa) long, of which we sequenced 750 bp (250 aa). In addition, European bison and wild boar GHR exon 10 were analysed using same methods and primers as described in [10]. Primer sequences are available from TI-T upon request.

In polymerase chain reaction (PCR), 50 ng of genomic DNA was used in 30 μl volume of standard DYNAZyme II (Finnzymes, Finland) PCR reaction mix. PCR products were purified using ExoSAP-IT enzyme (GE Healthcare Life sciences, UK). Sequencing reactions were performed with DYEnamic ET Terminator Kit (GE Healthcare Life sciences, UK). The sequencing products were purified with ethanol precipitation and separated on MegaBACE 1000 (Amersham Biosciences, UK). Each fragment was sequenced on both strands and same primers were used for the sequencing as for the fragment amplification. Sequence data was base-called with Cimarron 3.12 base-caller in program MegaBACE Sequence analyzer v. 3.0.0111.1603 (Amersham Biosciences, UK). All sequences were verified by visual inspection of chromatograms. Sequencher 4.6 (Gene Codes Corporation) was used to align and correct sequences.

### Statistical analysis

Haplotypes were reconstructed using the Bayesian haplotype reconstruction method for single nucleotide polymorphisms (SNP) from population genotype data utilizing the program PHASE v.2.1.1 [28]. Reconstructions were done using 10000 iterations, 1 thinning interval and 1000 as burn-in period assuming a stepwise mutation model. Animals that got haplotype pair probability lower than 0.95 were either cloned or discarded from further analysis. Cloning was done with Zero Blunt^® ^TOPO^® ^PCR Cloning Kit (Invitrogen, USA) and Phusion™ High-Fidelity DNA Polymerase (Finnzymes, Finland) following manufacturer's instructions. Five or more clones per individual were sequenced as described above using universal M13-primers. Haplotypes were decided according to the result obtained from the cloning.

We estimated nucleotide diversity, π (pi, [29]) and Watterson's theta estimator, θ_W _[30] along the exon 10 and made a sliding window plot from these two parameters among 18 vertebrate species. Pi and theta were calculated for 10 bp windows placed at 5 bp intervals using the following GenBank  sequences: NM_204854.1 (chicken), L76587.1 (turkey), XM_001508575.1 (platypus), NM_001039726.1 (cow), NM_000949.2 (human), NM_011169.4 (mouse), NM_001034111.1 (rat), NM_001082231.1 (European rabbit), XM_001500104.1 (horse), XM_536502.2 (dog), NM_001001868.1 (pig), NM_001085736.1 and NM_001085616.1 (Zenopus laevis), NM_001124599.1 (rainbow trout), DQ508436.1 (salmon), XM_001921376.1 (Danio rerio), NM_001078625.1 (takifugu), XM_001149377.1 (chimpanzee), and XM_001091532.1 (rhesus monkey).

We calculated haplotype diversity (H_d_), nucleotide diversity (pi) and Watterson's theta estimator for the studied species separately using haplotype sequences obtained. Pi is based on the average number of nucleotide differences between the sequences and theta is based on the total number of segregating sites in the sequence. To estimate the effect of selection, we calculated Tajima's D [31], and Fu and Li's D* and F* [32] for each species/subspecies separately. Tajima's D test compares the differences between the number of segregating sites and the average number of pairwise differences [31]. Under neutrality, Tajima's D value is assumed to be zero, under positive selection there is an excess of rare polymorphisms and Tajima's D value is negative. Negative D values can also be due to population expansion. If there is balancing selection intermediate frequency genetic variants are kept and Tajima's D value is positive. The same holds for the Fu and Li's D* and F* tests. Confidence intervals for the Tajima's D and Fu and Li's D* and F* values were obtained by generating 1000 independent coalescent simulations assuming no recombination. Statistical analysis package DnaSP 4.50.3 [33] was used to calculate H_d_, Pi, Watterson's theta estimator, Tajimas's D values and Fu and Li's D* and F*.

The impact of amino acid variants on protein structure via analysis of multiple sequence alignments was done with SIFT [13] and PolyPhen software [14]. SIFT uses sequence homology to predict whether an amino acid substitution will affect protein function and hence, potentially alter phenotype. It gives normalized probability score value that the amino acid change is tolerated. If score value is less than 0.05, amino acid change is predicted to be deleterious. Median conservation value for the diversity of the sequences in the alignment is measured as well. The default value is 3.0. Higher conservation values can lead to higher false positive error [13]. PolyPhen uses sequence alignments and protein 3D-structures for predictions. It does profile analysis of homologous sequences using BLAST search of the Non-Redundant DataBase (includes PDB sequences, SWISS-PROT, SWISS-PROTupdate, PIR, GenPept and GenPeptupdate). Sequence alignment is used by the PSIC (position-specific independent counts) software to calculate the profile matrix. Elements of the matrix are logarithmic ratios of the likelihood of a given amino acid occurring at a particular site to the likelihood of this amino acid occurring at any site. It computes also the absolute values of the difference between profile scores of both allelic variants in the polymorphic position. PolyPhen uses empirically derived rules to predict that an nsSNP is damaging or harmless i.e. most likely lacking phenotypic effect. No sequences of the intercellular or transmembrane part of PRLR are available for bison species and therefore analyses were done with the cytoplasmic part alone with these species. European and African cattle, pig and sheep were analyzed with the entire protein sequence.

Hardy-Weinberg equilibrium for each SNP, as well as linkage disequilibrium (LD as r^2 ^[34]) between PRLR exon 10 SNPs and between GHR and PRLR SNPs was calculated using Haploview 4.1 [35]. To generate a cladogram from the PRLR gene DNA sequences, we used statistical parsimony method [36] that finds the tree that requires the fewest evolutionary changes. This method is implemented for the TCS1.21 program [37].

## Authors' contributions

TI-T carried out the molecular genetic studies, performed the statistical analysis and drafted the manuscript. M-HL participated in molecular genetic studies and helped to draft the manuscript. ZG helped to draft the manuscript. JK and JV conceived of the study, and participated in its design and coordination and helped to draft the manuscript. All authors read and approved the final manuscript.

## References

[B1] Zeder MA, Emshwiller E, Smith BD, Bradley DG (2006). Documenting domestication: the intersection of genetics and archaeology. Trends Genet.

[B2] Zeder MA (2008). Domestication and early agriculture in the Mediterranean Basin: Origins, diffusion, and impact. Proc Natl Acad Sci USA.

[B3] Evershed RP, Payne S, Sherratt AG, Copley MS, Coolidge J, Urem-Kotsu D, Kotsakis K, Ozdogan M, Ozdogan AE, Nieuwenhuyse O, Akkermans PM, Bailey D, Andeescu RR, Campbell S, Farid S, Hodder I, Yalman N, Ozbasaran M, Bicakci E, Garfinkel Y, Levy T, Burton MM (2008). Earliest date for milk use in the Near East and southeastern Europe linked to cattle herding. Nature.

[B4] Andersson L, Georges M (2004). Domestic-animal genomics: deciphering the genetics of complex traits. Nat Rev Genet.

[B5] Gibbs RA, Taylor JF, Van Tassell CP, Barendse W, Eversole KA, Gill CA, Green RD, Hamernik DL, Kappes SM, Lien S, Matukumalli LK, McEwan JC, Nazareth LV, Schnabel RD, Weinstock GM, Wheeler DA, Ajmone-Marsan P, Boettcher PJ, Caetano AR, Garcia JF, Hanotte O, Mariani P, Skow LC, Sonstegard TS, Williams JL, Diallo B, Hailemariam L, Martinez ML, Morris CA, Silva LO, Spelman RJ, Mulatu W, Zhao K, Abbey CA, Agaba M, Araujo FR, Bunch RJ, Burton J, Gorni C, Olivier H, Harrison BE, Luff B, Machado MA, Mwakaya J, Plastow G, Sim W, Smith T, Thomas MB, Valentini A, Williams P, Womack J, Woolliams JA, Liu Y, Qin X, Worley KC, Gao C, Jiang H, Moore SS, Ren Y, Song XZ, Bustamante CD, Hernandez RD, Muzny DM, Patil S, San Lucas A, Fu Q, Kent MP, Vega R, Matukumalli A, McWilliam S, Sclep G, Bryc K, Choi J, Gao H, Grefenstette JJ, Murdoch B, Stella A, Villa-Angulo R, Wright M, Aerts J, Jann O, Negrini R, Goddard ME, Hayes BJ, Bradley DG, Barbosa da Silva M, Lau LP, Liu GE, Lynn DJ, Panzitta F, Dodds KG, Bovine HapMap Consortium (2009). Genome-wide survey of SNP variation uncovers the genetic structure of cattle breeds. Science.

[B6] Viitala S, Szyda J, Blott S, Schulman N, Lidauer M, Maki-Tanila A, Georges M, Vilkki J (2006). The role of the bovine growth hormone receptor and prolactin receptor genes in milk, fat and protein production in Finnish Ayrshire dairy cattle. Genetics.

[B7] Blott S, Kim JJ, Moisio S, Schmidt-Kuntzel A, Cornet A, Berzi P, Cambisano N, Ford C, Grisart B, Johnson D, Karim L, Simon P, Snell R, Spelman R, Wong J, Vilkki J, Georges M, Farnir F, Coppieters W (2003). Molecular dissection of a quantitative trait locus: a phenylalanine-to-tyrosine substitution in the transmembrane domain of the bovine growth hormone receptor is associated with a major effect on milk yield and composition. Genetics.

[B8] Bole-Feysot C, Goffin V, Edery M, Binart N, Kelly PA (1998). Prolactin (PRL) and its receptor: actions, signal transduction pathways and phenotypes observed in PRL receptor knockout mice. Endocr Rev.

[B9] Forsyth IA, Wallis M (2002). Growth hormone and prolactin–molecular and functional evolution. J Mammary Gland Biol Neoplasia.

[B10] Varvio SL, Iso-Touru T, Kantanen J, Viitala S, Tapio I, Maki-Tanila A, Zerabruk M, Vilkki J (2008). Molecular anatomy of the cytoplasmic domain of bovine growth hormone receptor, a quantitative trait locus. Proc Biol Sci.

[B11] Tomas A, Casellas J, Ramirez O, Munoz G, Noguera JL, Sanchez A (2006). High amino acid variation in the intracellular domain of the pig prolactin receptor (PRLR) and its relation to ovulation rate and piglet survival traits. J Anim Sci.

[B12] MacHugh DE, Shriver MD, Loftus RT, Cunningham P, Bradley DG (1997). Microsatellite DNA variation and the evolution, domestication and phylogeography of taurine and zebu cattle (Bos taurus and Bos indicus). Genetics.

[B13] Ng PC, Henikoff S (2003). SIFT: Predicting amino acid changes that affect protein function. Nucleic Acids Res.

[B14] Ramensky V, Bork P, Sunyaev S (2002). Human non-synonymous SNPs: server and survey. Nucleic Acids Res.

[B15] Larson G, Albarella U, Dobney K, Rowley-Conwy P, Schibler J, Tresset A, Vigne JD, Edwards CJ, Schlumbaum A, Dinu A, Balacsescu A, Dolman G, Tagliacozzo A, Manaseryan N, Miracle P, Van Wijngaarden-Bakker L, Masseti M, Bradley DG, Cooper A (2007). Ancient DNA, pig domestication, and the spread of the Neolithic into Europe. Proc Natl Acad Sci USA.

[B16] Kaplan NL, Darden T, Hudson RR (1988). The coalescent process in models with selection. Genetics.

[B17] Watterson GA, Guess HA (1977). Is the most frequent allele the oldest?. Theor Popul Biol.

[B18] Fang M, Larson G, Ribeiro HS, Li N, Andersson L (2009). Contrasting mode of evolution at a coat color locus in wild and domestic pigs. PLoS Genet.

[B19] Popadin K, Polishchuk LV, Mamirova L, Knorre D, Gunbin K (2007). Accumulation of slightly deleterious mutations in mitochondrial protein-coding genes of large versus small mammals. Proc Natl Acad Sci USA.

[B20] Tast A, Halli O, Ahlstrom S, Andersson H, Love RJ, Peltoniemi OA (2001). Seasonal alterations in circadian melatonin rhythms of the European wild boar and domestic gilt. J Pineal Res.

[B21] Linville RC, Pomp D, Johnson RK, Rothschild MF (2001). Candidate gene analysis for loci affecting litter size and ovulation rate in swine. J Anim Sci.

[B22] Lin CL, Ponsuksili S, Tholen E, Jennen DG, Schellander K, Wimmers K (2006). Candidate gene markers for sperm quality and fertility of boar. Anim Reprod Sci.

[B23] Harris J, Stanford PM, Oakes SR, Ormandy CJ (2004). Prolactin and the prolactin receptor: new targets of an old hormone. Ann Med.

[B24] Wallis M (1997). Function switching as a basis for bursts of rapid change during the evolution of pituitary growth hormone. J Mol Evol.

[B25] Kmiec M, Terman A (2006). Associations between the prolactin receptor gene polymorphism and reproductive traits of boars. J Appl Genet.

[B26] Roth T, Pfeiffer I, Weising K, Brenig B (2006). Application of bovine microsatellite markers for genetic diversity analysis of European bison (Bison bonasus). J Anim Breed Genet.

[B27] Miller SA, Dykes DD, Polesky HF (1988). A simple salting out procedure for extracting DNA from human nucleated cells. Nucleic Acids Res.

[B28] Stephens M, Smith NJ, Donnelly P (2001). A new statistical method for haplotype reconstruction from population data. Am J Hum Genet.

[B29] Nei M (1987). Molecular Evolutionary Genetics.

[B30] Watterson GA (1975). On the number of segregating sites in genetical models without recombination. Theor Popul Biol.

[B31] Tajima F (1989). Statistical method for testing the neutral mutation hypothesis by DNA polymorphism. Genetics.

[B32] Fu YX, Li WH (1993). Statistical tests of neutrality of mutations. Genetics.

[B33] Rozas J, Sanchez-DelBarrio JC, Messeguer X, Rozas R (2003). DnaSP, DNA polymorphism analyses by the coalescent and other methods. Bioinformatics.

[B34] Hill WG (1975). Linkage disequilibrium among multiple neutral alleles produced by mutation in finite population. Theor Popul Biol.

[B35] Barrett JC, Fry B, Maller J, Daly MJ (2005). Haploview: analysis and visualization of LD and haplotype maps. Bioinformatics.

[B36] Templeton AR, Crandall KA, Sing CF (1992). A cladistic analysis of phenotypic associations with haplotypes inferred from restriction endonuclease mapping and DNA sequence data. III. Cladogram estimation. Genetics.

[B37] Clement M, Posada D, Crandall KA (2000). TCS: a computer program to estimate gene genealogies. Mol Ecol.

